# Herbicide Resistance Is Increasing in Spain: Concomitant Management and Prevention

**DOI:** 10.3390/plants12030469

**Published:** 2023-01-19

**Authors:** José María Montull, Joel Torra

**Affiliations:** 1 Grupo de investigación en Malherbología y Ecología Vegetal, Departamento de Hortofruticultura, Botánica y Jardineria, ETSEA, Agrotecnio-CERCA Center, Universidad de Lleida, 25198 Lleida, Spain; 2 Comité de Prevención de las Resistencias a los Herbicidas (CPRH), Working Group of the Spanish Weed Science Society (Sociedad Española de Malherbología, SEMh), 50059 Zaragoza, Spain

**Keywords:** *Amaranthus palmeri*, annual summer grass weeds, *Bassia scoparia*, *Bromus*, *Conyza*, *Lolium*, *Salsola kali*

## Abstract

Herbicide-resistant weeds currently challenge sustainable food production in almost all cropping systems in Europe. Herbicide resistance is increasing, and some European countries are among the most affected globally, such as Spain and France. This situation is worsening not only due to herbicide use restrictions but also due to climate change, rendering Mediterranean countries such as Spain particularly susceptible. Therefore, focus should be aimed at preventive measures, which include those not only based on integrated weed management strategies but also based on a very good knowledge of the biology and ecology of each weed species. The main objective of this review is to provide an overview of potential future herbicide-resistant cases that can evolve in the near future in Europe. We use Spain as the case study, as it is the most affected country in Europe and because it is at risk due to global warming. For different resistant cases detailed on a crop basis, adequate prevention and management measures will be provided in order to avoid resistance evolution relative to the sites of action that are most likely to generate resistant biotypes due to expected high selection pressures.

## 1. Introduction

Herbicide-resistant weeds are expected to continue to grow and ultimately threaten crop production on a global basis [1]. Unfortunately, Spain (with around 500,000 km^2^) with 23 Mha of arable land, in the Mediterranean southwestern corner of Europe, is not an exception. Spain ranks sixth in the world, and second in Europe (after France) in terms of unique herbicide-resistant weed cases [2]. According to the updated information provided in a recent review, it now ranks first in Europe, and fifth in the world, which is right behind the largest countries in the world in terms of both food production and surface area: Canada, USA, Brazil, and Australia [3].

Resistance managements should start with the implementation of integrated weed management (IWM) strategies to reduce the selection pressure and reliance on herbicides [4]. The best IWM measure against herbicide resistance is diversification at all levels: diverse cropping systems or crop rotations, a variety of control methods (non-chemical and chemical), and increasing crop density and delayed seeding [5]. It is acknowledged that all weed control methods, even hand weeding, can promote the evolution of resistance traits in field weed populations, which is why diversity at all levels is crucial.

From a chemical perspective, using herbicides with different Sites of Action (SoAs) in tank mixtures, annual rotations, and sequential applications can delay the evolution of herbicide resistance by reducing the selection pressure imposed on weeds by a single herbicide SoA [4]. In Europe, because of the scarcity of new products in the pipeline and the withdrawal or mandatory dose rate reduction in many active ingredients resulting from the re-registration process, there are limited options allowing the rotation or tank mixing of active ingredients. Moreover, some active ingredients with high environmental toxicity such as pendimethalin or prosulfocarb are applied in several crops into the rotation due to the limitation of new approvals. In addition, there are some weed control strategies based on herbicide-tolerant crops with respect to acetolactate synthase (ALS) or acetil-CoA carboxylase ACCase inhibitors, such as sunflower, oilseed rape, rice, and sugar beet. This increases selection pressure over these chemistries and the risks of further selecting resistant biotypes [6].

Furthermore, any specific spot of plants surviving an herbicide application should be treated as a putative resistance case, and these populations should be managed so that survivors do not shed any seed that could enter the seed bank. Consequently, over the last years, the most relevant change in strategy in herbicide-resistant weed management globally has been the increased attention on reducing the weed seed bank and maintaining low seed bank levels by using any tactic [7]. At the field level, farmers or stakeholders do not notice these phenomena and set management measures until more or less 30% of the plants within a weed population are resistant, which is typically too late. This is why the early detection of herbicide resistance is a key prevention measure that can enable a timely response [4].

The main objective of this review is to provide an overview of potential future herbicide-resistant cases that can evolve in the near future in Europe, and Spain is used as a case study, as it is the most affected country in Europe and has a high diversity of crops and agro-climatic conditions. For the different resistant cases detailed on a crop basis, adequate prevention and management measures will be proposed to avoid resistance evolutions on the most threatened sites of action due to expected high selection pressures. Most herbicide resistance cases that will be detailed in this review have yet been confirmed in Spain or even Europe, so the management guidelines provided should delay the evolution of resistance. However, the spread of resistant biotypes via contamination (of seed, commodities, machinery, etc.) will require additional measures.

## 2. Herbicide-Resistant Cases in Perennial Crops

Glyphosate (Group 9, HRAC legacy group G) is currently the only non-selective post-emergence herbicide registered in Europe [3]. In perennial woody crops, it is still the main (chemical) weed control tool used to manage weeds both under crop plants and along inter-rows [8]. Therefore, it is expected that the high selection pressure with this herbicide will continue or probably will even increase in different cropping systems across the continent. Spain represents a good example of the worsening scenario for perennial crops in Europe. Until the beginning of the 21st century, no cases of glyphosate were reported in this country. However, since then, with the first report for *Conyza bonariensis*, there has been a fast and steady increase in the number of cases at up to 18, particularly in perennial crops [3]. Unfortunately, the main response to glyphosate resistance has been to switch to another SoA to control glyphosate-resistant populations. Therefore, one of the expected outcomes of this improper management is the evolution of multiple herbicide-resistant weeds in different European perennial crops. In fact, multiple herbicide-resistant populations have already been confirmed in perennial crops from Spain for *Lolium* and *Conyza* species [9,10], which will be treated in depth here (Table 1).

### 2.1. Genus Conyza

The genus *Conyza* includes around 100 species distributed all over the world and they are not only observed mainly in tropical climates but also in temperate areas of the Northern Hemisphere [11]. The main weeds species present in Europe are *C. bonariensis*, *C. canadensis*, and *C. sumatrensis*, which are commonly known as fleabanes. The three species are natives of the American continent, and *C. bonariensis* and *C. sumatrensis* are from South America, while *C. canadensis* is from from North America [12].

These species of the *Conyza* genus are well known for their prominence as weeds, particularly under the rows of perennial crops, where they limit the presence of other weed species and grow with limited competition [12]. Besides these biological attributes, they have natural reduced susceptibility relative to glyphosate at later growth stages together with being prone to evolve resistance relative to this non-selective herbicide [13].

Until very recently, the use of oxyfluorfen (Group 14, HRAC legacy group E) to residually control broadleaf weeds and also some grasses was common in perennial crops [9]. In general, herbicides inhibiting protoporphyrinogen oxidase (PPO), such as oxyfluorfen, are at low risks of resistance evolution in weeds [14,15]. However, due to environmental concerns, the registered dose in Europe for oxyfluorfen has been reduced, so its effectiveness and use is now limited to post-emergence applications [16]. This scenario seriously increased the dependency on other herbicide SoAs, mainly ALS-inhibiting herbicides (Group 2, HRAC legacy group B) such as flazasulfuron, iodosulfuron, and penoxsulam in perennial crops such as citrus, olive, and vineyards. Additionally, these *Conyza* species have the capacity to evolve resistance relative to ALS inhibitors, with several confirmed cases across the globe [2]. For example, glyphosate-resistant populations were managed with post-emergence ALS inhibitors, i.e., prompting the evolution of multiple herbicide-resistant *C. canadensis* to both SoAs in Spanish olive orchards [17]. In the worst cases, farmers switch from one SoA to another to manage resistant populations. A *C. bonariensis*-resistant biotype to at least five SoAs is already present in Spanish olive fields [10].

In susceptible populations to glyphosate, it would be crucial to avoid seed production from any plant surviving the application of ALS-inhibiting herbicides. In this case, a possibility is the mixture of the ALS-inhibiting herbicide with 2,4-D or other auxinic herbicides in order to improve effectiveness. However, in populations that are already resistant, it is very difficult to adequately decide upon the timing of post-emergence contact herbicides, hindering their management only by chemical means. Therefore, the only way to alleviate the evolution herbicide resistant in *Conyza* species is to implement non-chemical and cultural methods to manage them in perennial crops. These should include a mixture of different modes of action (MoA), mechanical control (mowing and shredding), or cover crops among others. Finally, organic mulches under crop plants or bioherbicides are promising new tools to consider for managing *Conyza* [18,19].

### 2.2. Genus Lolium

The species that belong to the genus *Lolium* and particularly *L. rigidum* are among the worst herbicide-resistant weeds globally. Multiple herbicide-resistant populations are common in different cropping systems [2]. Currently, *L. rigidum* is the most worrying and important herbicide-resistant weed species in Mediterranean Europe [3]. Oxyfluorfen, alone or mixed with glufosinate (Group 10, HRAC legacy group H) and glyphosate, were not only applied to control grass and broadleaf weeds in perennial crops but also to prevent and/or control glyphosate-resistant *Lolium* spp. populations [9,20] with the risk of multiple herbicide resistance evolution. However, in the EU, glufosinate was banned in 2014 and the actual authorized dose rate of oxyfluorfen does not adequately control *L. rigidum*.

In perennial crops, the main factor that hinders the chemical management of *Lolium* spp. is the evolution of biotypes with multiple resistance to ALS inhibitors and glyphosate. On a chemical basis, the only way to control these biotypes at the seedling stage is the use of ACCase-inhibiting herbicides (Group 1, HRAC legacy group A), such as propaquizafop, which implies that the selection pressure on this SoA will be very high. In fact, resistance to ACCase inhibitors is the most common case in European winter cereals for *L. rigidum* [2], stressing the facility to evolve resistance to this SoA in these species [21]. The evolution of multiple herbicide resistance with respect to glyphosate, ACCase inhibitors, and other SoAs (oxyfluorfen and ALS inhibitors) is expected in Mediterranean countries such as Spain. For these reasons, resistant cases can start to appear in *Lolium* spp. after only three to four seasons, and these biotypes are almost impossible to manage by chemical means [22]. In the worst cases of multiple resistance, the tolerance to seed production should be zero for areas where herbicides are failing. It is important to highlight that the seed bank persistence in the soil for *Lolium* species is relatively low compared to other weeds at usually 2–3 years; thus, with adequate weed management, including mowing and non-chemical methods (Table 1), the resistant population should not spread.

## 3. Herbicide-Resistant Cases in Winter Annual Crops

Winter annual crops represent the most extensive cultivated area in Europe at more than 40 × 10^6^ ha. Weeds comprise the main yield constraints [23], and they are still mainly managed using herbicides; therefore, due to so many years of their use in monocrops, they are one of the most affected crops in European cropping systems with respect to resistant weeds [2]. Historically, first reported cases were resistance to ACCase inhibitors in several grasses, with subsequent cases of single resistance or multiple resistance relative to ALS inhibitors. Winter annual crops are still highly dependent on ALS inhibitors in part due to due to ACCase inhibitors’ resistance, although these cropping systems tend to be more diverse in Europe compared to other continents, with an increase in oilseed rape, winter peas, winter beans, and spring barley [1]. The evolution of (multiple) resistance to ACCase and ALS-inhibiting herbicides in grass weeds in winter annual crops prompted an increase in the use of pre-emergence herbicides [1]. This chemical management operation risks facilitating the appearance of multiple herbicide-resistant weeds relative to three or more SoAs (ACCase, ALS, and other inhibitors), as exemplified in Mediterranean Europe by *L. rigidum*, with populations resistant toward ACCase, ALS, and photosystem II (PS II) inhibitors being common [24] or populations resistant toward at least four SoAs (ACCase, ALS, PS II, and very-long-chain fatty acid synthase (VLCFAS) inhibitors) spreading [25] in Spain.

In a European context of herbicide dose reduction, particularly of residual herbicides from the re-registration process, a limited number of available SoAs, and few new ones that are incoming, resistance in winter annual crops is expected to increase in the future [1,3]. The biggest issues faced include simplified agronomic practices such as monoculture and the use of herbicides with the same SoA, as ACCase and ALS inhibitors are still predominant. Therefore, multiple herbicide-resistant weeds relative to selective SoAs will increase, raising the selection pressure to non-selective herbicides (glyphosate) in annual crops under no-till farming due to an increase in seeds in the seedbank with the concomitant evolution of glyphosate-resistant weeds. Since the most important weed species in winter annual crops—*L. rigidum* among grasses and *Papaver rhoeas* among broadleaf weeds—are reviewed in the previous literature [3], potential new and distinct resistant cases are treated below.

### 3.1. Genus Bromus

Native from the Mediterranean region, the *Bromus* species have become one of the most problematic weeds in Europe and Spain because of the monoculture of winter cereals under minimal no-till farming and the difficulty in controlling (lack of efficient active ingredients) them with selective herbicides [26]. These species, such as *B. diandrus*, were minor weeds in the past, but as of the mid-1970s, there is a common awareness that the *Bromus* spp. problem worsened, which is what occurred with *B. sterilis* in the United Kingdom [27] or *B. diandrus* and *B. madritensis* in Spain [28,29].

The weed species belonging to the *Bromus* genus spread year after year in different cropping systems under conservation agriculture [30], even when intensive and conventional tillage processes occur [28]. This scenario implies a higher selection pressure with selective herbicides for their control. This chemical management is mainly achieved with ALS inhibitors in wheat mesosulfuron, pyroxsulam, or propoxycarbazone. Therefore, there is a high risk of selecting herbicide-resistant populations for this SoA in the short term. Resistance toward ALS inhibitors has been reported for *B. sterilis* in several European countries [2].

The *Bromus* species, such as *B. diandrus*, have a short emergence period in autumn [28]. For these reasons, several studies demonstrated the usefulness of delayed seeding: for example, rotating cereals with legume crops sown from December combined with the use of ACCase inhibitors, as a good strategy to manage these species [28,31]. These strategies combined with other non-chemical and cultural practices (Table 1), coupled with the low persistence of seeds in the soil seed bank, should facilitate a safe and sustainable management of *Bromus* spp. in winter cereals. Finally, using harvest weed seed control (HWSC) tactics has potential in effectively managing *Bromus* species [32]. HWSC is based on a zero tolerance of seed production or surviving weeds at harvest.

### 3.2. Other Winter Grass Weeds

Several species belonging to both the genera *Avena* and *Phalaris* have a high risk of evolving resistance relative to post-emergence selective herbicides, such as ACCase inhibitors. Unfortunately, resistant cases in both genera toward this SoA were already reported in some European countries [15]. Both genera have staggered flushes of emergences from autumn to spring, hindering decision making and, in general, prompting late post-emergence herbicide applications. Additionally, some of the crops used in rotations are usually managed with the same SoAs, increasing the risk of herbicide-resistant evolution. Particularly for the *Avena* species, controlling them with pre-emergence herbicides results in very low to null effects, because their seeds can emerge from deep soil layers; thus, the selection pressure by post-emergence contact herbicides, such ACCase and ALS inhibitors, is even higher. In Spain, (multiple) herbicide-resistant biotypes to these two SoAs were reported in *A. sterilis* ssp. *ludoviciana* and *A. fatua* from winter cereals [33]. Therefore, IWM programs for managing this herbicide-resistant grasses should consider, from a chemical basis, the mixture, sequence, and rotation of different MoAs and, among cultural tactics, crop rotation and the variation in sowing dates (Table 1).

### 3.3. Amaranthaceae: Bassiascoparia and Salsola kali

Kochia (*Bassia scoparia*) and prickly Russian thistle (*Salsola kali*) belong to the Amaranthaceae family (previously in the Chenopodiaceae family) after reclassification [34,35]. These two species are becoming increasingly frequent in winter cereals fields from Spain, and they can be observed all summer until the beginning of the next season in early autumn when they reach the maturity; moreover, the seeds released by wind or parent plants are transported by wind as tumbleweeds, spreading their diaspores [36].

*B. scoparia* and *S. kali* usually germinate in early spring, but the emergence period can span from February to June depending on the region [37,38]. Climate change is probably incresaing their emergence timespan and mainly advancing it to later winter, due to warmer temperatures, particularly in a Mediterranean climate [38]. These two species are generally not problematic within the crop season in winter cereals because their growth under the crop canopy is quite limited and selective herbicides are still efficient. Within crops, they are usually controlled with ALS-inhibiting herbicides because they can be applied until the flag leaf growth stage of cereals. These species can be troublesome in sugar beet, with a worsening scenario due to the appearance of the first resistant cases to ALS inhibitors in the province of Valladolid [39]. Moreover, both species are weakly susceptible to residual herbicides such as pendimethalin, which leads to limited weed control in broadleaf crops such as legumes, one of the main rotational crops in winter annual cropping systems. In these rotational crops such as legumes or Clearfield oilseed rape, the use of imazamox introduces a risk of resistance toward ALS inhibitors.

This scenario is not only increasing the selection pressure by ALS-inhibiting herbicides during the crop season but also by glyphosate and synthetic auxin herbicides after cereal harvest during the summer fallow. Therefore, there is a high risk of selecting herbicide-resistant populations to these SoAs in both species, as demonstrated by the presence of these biotypes in large cereal-producing areas from the USA, Canada, and Australia [2]. In the Mediterranean cereal-producing areas of Europe, such as in Spain, these resistant biotypes can also evolve if non-chemical and cultural methods are not integrated for their management, such as the use of a shredder during the summer fallow period or occasional tillage [40].

## 4. Herbicide-Resistant Cases in Summer Annual Crops

In the last two decades, weed management in annual summer crops in Europe is becoming more challenging, particularly in maize. The main reason is the appearance of herbicide-resistant grass weeds toward ALS-inhibiting herbicides [2]. The scenario was already difficult since this SoA is one of the few available options for managing some grass weeds in post-emergence maize. An extreme case is *Sorghum halepense*, because its chemical control completely relies on ALS inhibitors in corn. Currently, several herbicide-resistant grass species have been reported in Europe in different summer annual crops, such as *Echinochloa crus-galli*, *S. halepense*, or *Setaria*, particularly relative to ALS inhibitors [2]. The restricted use of terbuthylazine, which is obligatory since 2021 [41], complicates the management of these annual grass weeds even more, as provided by residual activities and additional effects relative to chloroacetamide such as S-metolachlor. Terbuthylazine also provides good control for the Amaranth species and other broadleaved weeds. This restriction will also increase the selection pressure over HPPDs, ALS, and Auxinics to control broadleaved weed species.

### 4.1. Summer Annul Grasses

Several grass weed species, such as *E.cruss-galli*, *Digitaria sanguinalis*, *Panicum dichotomiflorum*, or *Setaria* spp., frequently appear both in corn fields and other annual summer crops: sunflower, sorghum, soybean, sugar beet, potatoes, and tomatoes. For some of these species, resistance to ALS-inhibiting herbicides (nicosulfuron or rimsulfuron) has already been reported in Spain, namely *E. crus-galli* [42] or *S. halepense* [43]. For this reason, the tendency in maize crop is to increase the frequency of the use of residual herbicides such as chloroacetamides and triketones to control them. Both terbuthylazine and chloroacetamides are being re-evaluated to limit their use in Europe due to the contamination of soil reservoirs of drinking water. In fact, during the summer of 2022, there was a shortage of drinking water in some maize-producing areas in Catalonia due to contamination with S-metolachlor (chloroacetamide) and terbuthylazine. For this reason, the use of these and other residual herbicides should be optimized to secure their long-term sustainability. Decision support tools such as IPMwise should be used to attain this goal. IPMWise is a DSS that optimizes active ingredients and dosages according to the actual weed species and spraying conditions for each specific field. As an average of four countries, herbicide average savings between 20 and 50% can be expected for maintaining the efficacy levels, and it can be used to generate variable rate spraying maps [44]. It is important to highlight that, for the majority of crops, chloroacetamides are the key active ingredients that have pre-emergence and can residually control annual grass weeds. If its use is restricted or banned in Europe, the selection pressure with ACCase-inhibiting herbicides will increase, which are used in broadleaf rotational crops with maize. It is known that this SoA is most prone toward selecting herbicide-resistant grass weeds; in fact, several cases in annual summer crops were reported across different European countries [2]. 

A particular and relevant example is the operations involved in processing tomato crops, because there are only three registered SoAs: only S-metolachlor among VLCFAS inhibitors in pre-emergence and rimsulfuron (ALS inhibitor) and ACCase inhibitors in post-emergence. Therefore, in a scenario of S-metolachlor restriction or banning combined with the appearance of ALS-inhibitor-resistant populations, all chemical control for managing annual grass weeds would rely on ACCase inhibitors, with a concomitant risk of selecting multiple herbicide-resistant populations to both SoAs.

The non-chemical and cultural methods for managing these weed species should include crop rotation, changing sowing dates, and also different MoAs mixtures, sequences, and rotations. The potential use of HWSC strategies should also be researched.

### 4.2. Amaranthus palmeri

Special attention should be devoted to a new invasive weed species in summer crops in Europe: Palmer amaranth (*Amaranthus palmeri*). *A. palmeri* is present mainly in Italian soybean fields [45] and maize fields and roadsides in Spain [46]. The main biological attributes that explain their noxiousness are the following: extreme fecundity, very high competitive ability due to fast growth, and high genetic variability [47]. Moreover, the arrival or selection of herbicide-resistant biotypes is hindering its potential management in Europe both in crop and non-crop lands. Resistance to ALS-inhibiting herbicides has been confirmed in Italy and Spain [45,46], while resistance to glyphosate has recently been reported in roadside populations in Spain [48].

Therefore, post-emergence control is at risk, which will increase selection pressures by pre-emergence and residual herbicides and by the few available post-emergence options, such as synthetic auxin herbicides. The European context of herbicide dose reduction, particularly of pre-emergence herbicides, from the re-registration process will facilitate this scenario because more surviving plants after pre-emergence treatments will increase selection pressures over post-emergence treatments. If the required prevention and curative measures are not undertaken, the evolution of resistance to pre-emergence herbicides or auxin mimics will occur in the near future. These resistance cases have already been reported in several populations in North America [2]. For example, it is known that three applications of dicamba at sublethal doses are sufficient for promoting the evolution of resistance in *A. palmeri* [49]. Besides eradication programs, which were already undertaken in Spain [3], on a chemical basis, the mixture of at least two SoAs, both in pre- and post-emergence and the sequential pre- and post-treatments (also SoAs mixtures) are mandatory for controlling this invasive weed species. However, of course, the use of IWM programs that include non-chemical and cultural strategies is crucial (Table 1) in order to alleviate herbicide selection pressure and to reduce our dependency on chemical management. Among these tactics, implementing HWSC control tactics to reduce the risk of evolution resistance has potential [50].

## 5. Concluding Remarks

Overall, among all the studied cases, the restriction in the use of some active ingredients promotes a higher use of those that are still under registration. This fact implies a lower diversification of the chemical weed control tools, increasing the risk of rapid herbicide resistance evolution due to a higher selection pressure exerted by the herbicides that are still available and effective. Unfortunately, weed herbicide resistance is an increasing reality observed in European crops. The complexity of the scenario is becoming worse, and the management and mostly the prevention are mandatory from a multidisciplinary perspective that integrates all of the available knowledge for each weed species. In this article, the potential herbicide resistance evolution in important weed species in Europe has been reviewed, placing the focus on prevention strategies to delay this evolutionary process as much as possible. One of the potential solutions for alleviating the selection pressure and dependency on herbicides is the incorporation of new control technologies. One of the best examples include harvest weed seed control (HWSC) tools, which help to reduce herbicide use and manage resistance in the long-term period, as already mentioned several times. Despite its potential, there is still minor adoption of HWSC in Europe [51].

## Figures and Tables

**Table 1:** Crop type, modes of action (MoA) at risk (resistance/registration), species or genus at risk of resistance evolution, and best integrated weed management (IWM) strategies to limit the spread of resistance.

Crop Type	MoA *	Species/Genus	IWM Strategies †
Perennial woody	EPSPS, PPO	*Conyza*	MoA mixture, mowing, and shredding
	ALS, ACCase	*Lolium*	avoid seed shed and cover crops
	SAH		mulchings and bioherbicides
Winter annual	EPSPS, SAH	*Bromus*	Crop rotation and sowing date
	ACCase, ALS	*Avena*	MoA mixture, sequence, and rotation
	PS II, VLCFAS	*Phalaris*	Mechanical control in fallow (tillage and shredding)
		*Bassia scoparia*	HWSC
		*Salsola kali*	
Summer annual	ALS, PS II	*Digitaria sanguinalis*	Crop rotation, sowing date
	ACCase, HPPD	*Panicum dichotomiflorum*	MoA mixture, sequence, and rotation
	VLCFAS	*Setaria*	HWSC
		*Amaranthus palmeri*	

* Target protein and group. EPSPS: 5-enolpyruvylshikimate-3-phosphate synthase, 9; PPO: protoporphyrinogen oxidase, 14; ALS: acetolactate synthase, 2; ACCase: acetyl-CoA carboxylase, 1; SAH: synthetic auxin herbicides, 4; PS II: photosystem IID1 Serine 264 binders, 5; VLCFAS: very long chain fatty acid synthesis, 15; HPPD: 4-Hydroxyphenylpyruvate dioxygenase, 27. † HWSC: harvest weed seed control: seed terminator.

## Data Availability

Not applicable.

## References

[1] Peterson M.A., Collavo A., Ovejero R., Shivrain V., Walsh M.J. (2018). The challenge of herbicide resistance around the world: A current summary. Pest Manag. Sci..

[2] Heap I. The International Herbicide-Resistant Weed Database. https://www.weedscience.org.

[3] Torra J., Montull J.M., Calha I.M., Osuna M.D., Portugal J., de Prado R. (2022). Current Status of Herbicide Resistance in the Iberian Peninsula: Future Trends and Challenges. Agronomy.

[4] Norsworthy J., Ward S., Shaw D., Llewellyn R., Nichols R., Webster T., Bradley K.W., Frisvold G., Powles S.B., Burgos N.R. (2012). Reducing the Risks of Herbicide Resistance: Best Management Practices and Recommendations. Weed Sci..

[5] Liebman M., Staver C.P., Liebman M., Mohler C.L., Staver C.P. (2001). Crop diversification for weed management. Ecological Management of Agricultural Weeds.

[6] Lobman A., Christen O., Petersen J. (2019). Development of herbicide resistance in weeds in a crop rotation with ALS-tolerant sugar beets under vaying selection presure. Weed Res..

[7] Beckie H.J., Ashworth M.B., Flower K.C. (2019). Herbicide Resistance Management: Recent Developments and Trends. Plants.

[8] Duke S.O. (2018). Glyphosate: The world’s most successful herbicide under intense scientific scrutiny. Pest Manag. Sci..

[9] Fernández P., Alcántara R., Osuna M.D., Vila-Aiub M.M., De Prado R. (2016). Forward selection for multiple resistance across the non-selective glyphosate, glufosinate and oxyfluorfen herbicides in *Lolium* weed species. Pest Manag. Sci..

[10] Palma-Bautista C., Vázquez-Garciá J.G., Domínguez-Valenzuela J.A., Ferreira Mendes K., Alcántara De La Cruz R., Torra J., De Prado R. (2021). Non-target-site resistance mechanisms endow multiple herbicide resistance to five mechanisms of action in *Conyza bonariensis*. J. Agric. Food Chem..

[11] Noyes R.D. (2000). Biogeographical and evolutionary insights on Erigeron and allies (Asteraceae) from ITS sequence data. Plant Syst. Evol..

[12] Florentine S., Humphries T., Chauhan B.S., Chauhan B.S. (2021). Chapter 7—*Erigeron bonariensis*, *Erigeron canadensis*, and *Erigeron sumatrensis*. Biology and Management of Problematic Crop Weed Species.

[13] Amaro-Blanco I., Fernández-Moreno P.T., Osuna-Ruiz M.D., Bastida F., De Prado R. (2018). Mechanisms of glyphosate resistance and response to alternative herbicide-based management in populations of the three *Conyza* species introduced in southern Spain. Pest Manag. Sci..

[14] Powles S.B., Yu Q. (2010). Evolution in action: Plants resistant to herbicides. Annu. Rev. Plant Biol..

[15] Riggins C.W., Tranel P.J. (2012). Will the *Amaranthus tuberculatus* Resistance Mechanism to PPO-Inhibiting Herbicides Evolve in Other *Amaranthus* Species?. Int. J. Agron..

[16] European Commission (EC) (2017). Commission Implementing Regulation (EU) 2017/359 of 28 February 2017 amending Implementing Regulation (EU) No 540/2011 as Regards the Conditions of Approval of the Active Substance Oxyfluorfen. https://eur-lex.europa.eu/legal-content/EN/TXT/?uri=uriserv:OJ.L_.2017.054.01.0008.01.ENG.

[17] Mora D.A., Cheimona N., Palma-Bautista C., Rojano-Delgado A.M., Osuna-Ruiz M.D., de la Cruz R.A., De Prado R. (2019). Physiological, biochemical and molecular bases of resistance to tribenuron-methyl and glyphosate in *Conyza canadensis* from olive groves in southern Spain. Plant Physiol. Biochem..

[18] Cabrera-Pérez C., Valencia-Gredilla F., Royo-Esnal A., Recasens J. (2022). Organic Mulches as an Alternative to Conventional Under-Vine Weed Management in Mediterranean Irrigated Vineyards. Plants.

[19] Cabrera-Pérez C., Royo-Esnal A., Recasens J. (2022). Herbicidal Effect of Different Alternative Compounds to Control *Conyzabonariensis* in Vineyards. Agronomy.

[20] Fernández-Moreno P.T., Travlos I., Brants I., De Prado R. (2017). Different levels of glyphosate-resistant *Lolium rigidum* L. among major crops in southern Spain and France. Sci. Rep..

[21] Vázquez-García J.G., Alcántara-de la Cruz R., Palma-Bautista C., Rojano-Delgado A.M., Cruz-Hipólito H.E., Torra J., Barro F., De Prado R. (2020). Accumulation of Target Gene Mutations Confers Multiple Resistance to ALS, ACCase, and EPSPS Inhibitors in *Lolium* Species in Chile. Front. Plant Sci..

[22] Busi R., Gaines T.A., Walsh M.J., Powles S.B. (2012). Understanding the potential for resistance evolution to the new herbicide pyroxasulfone: Field selection at high doses versus recurrent selection at low doses. Weed Res..

[23] Oerke E.C. (2006). Crop losses to pests. J. Agric. Sci..

[24] Loureiro I., Escorial C., Plaza E.H., Gonzalez-Andujar J.L., Chueca M.C. (2017). Current status in herbicide resistance in *Loliumrigidum* in winter cereal fields in Spain: Evolution of resistance 12 years after. Crop Prot..

[25] Torra J., Montull J.M., Taberner A., Onkokesung N., Boonham N., Edwards R. (2021). Target-Site and Non-target-Site Resistance Mechanisms Confer Multiple and Cross- Resistance to ALS and ACCase Inhibiting Herbicides in *Loliumrigidum* from Spain. Front. Plant Sci..

[26] Escorial C., Loureiro I., Rodríguez-García E., Chueca C. (2011). Population variability in the response of ripgut brome (*Bromusdiandrus*) to sulfosulfuron and glyphosate herbicides. Weed Sci..

[27] Cussans G.W., Cooper F.B., Davies D.H.K., Thomas M.R. (1994). A survey of the incidence of the *Bromus* species as weeds of winter cereals in England, Wales and parts of Scotland. Weed Res..

[28] Recasens J., García A.L., Cantero-Martínez C., Torra J., Royo-Esnal A. (2016). Long-term effect of different tillage systems on the emergence and demography of *Bromusdiandrus* in rainfed cereal fields. Weed Res..

[29] Vázquez-García J.G., Castro P., Royo-Esnal A., Palma-Bautista C., Torra J., De Prado R. (2023). First report of a wide distribution of glyphosate resistant *Bromus madritensis* L. in the Iberian Peninsula: Confirmation and field management. Weed Sci..

[30] Borger C.P.D., Torra J., Royo-Esnal A., Davies L., Newcombe G., Chauhan B.S. (2021). Chapter 4—*Bromus diandrus* and *Bromus rigidus*. Biology and Management of Problematic Crop Weed Species.

[31] Royo-Esnal A., Recasens J., Garrido J., Torra J. (2018). Rigput Brome (*Bromusdiandrus* Roth.) Management in a No-Till Field in Spain. Agronomy.

[32] Borger C.P.D., Petersen D., Gill G.S. (2021). Modelling the long-term impact of harvest weed seed control for species like *Bromus diandrus* and *Hordeum* spp. that shed a portion of seed prior to harvest. Weed Res..

[33] Torra J. (2022). Personal communication.

[34] Müller K., Borsch T. (2005). Phylogenetics of Amaranthaceae Based on matK/trnK Sequence Data: Evidence from Parsimony, Likelihood, and Bayesian Analyses. Ann. Missouri Bot. Gard..

[35] The Plant List Version 1.1. http://www.theplantlist.org/.

[36] Baker D., Beck K., Bienkiewicz B., Bjostad L. (2008). Forces Necessary to Initiate Dispersal for Three Tumbleweeds. Invasive Plant Sci. Manag..

[37] Kumar V., Jha P., Dille J., Stahlman P. (2018). Emergence Dynamics of Kochia (*Kochia scoparia*) Populations from the U.S. Great Plains: A Multi-Site-Year Study. Weed Sci..

[38] Santín-Montanyá M.I., Gandía M.L., Casanova C., Sánchez-Jiménez F.J., Tenorio J.L. (2020). The influence of soil tillage system on *Salsola kali* L. emergence during the fallow period within cereal fields. Soil Use Manag..

[39] Comité Prevención Resistencia Herbicidas (CPRH) (2022). Working Group of the Spanish Weed Science Society.

[40] Schillinger W. (2007). Ecology and Control of Russian Thistle (*Salsola iberica*) after Spring Wheat Harvest. Weed Sci..

[41] European Commission (EC) (2022). Commission Implementing Regulation (EU) 2021/824 of 21 May 2021 Amending Implementing Regulations (EU) No 540/2011 and (EU) No 820/2011 as Regards the Conditions of Approval of the Active Substance Terbuthylazine. https://eur-lex.europa.eu/legal-content/EN/TXT/?uri=CELEX%3A32021R0824.

[42] Torra J., Montull J.M., Royo-Esnal A., Taberner A., Salas M.L. Resistance to ALS inhibiting herbicides in a Spanish *Echinochloa cruss-galli* population from a corn field. Proceedings of the Conference: Resistance’19, Poster 33.

[43] Travlos I.S., Montull J.M., Kukorelli G., Malidza G., Dogan M.N., Cheimona N., Antonopoulos N., Kanatas P.J., Zannopoulos S., Peteinatos G. (2019). Key Aspects on the Biology, Ecology and Impacts of Johnsongrass [*Sorghum halepense* (L.) Pers] and the Role of Glyphosate and Non-Chemical Alternative Practices for the Management of This Weed in Europe. Agronomy.

[44] Montull J.M., Taberner A., Bojer O., Rydahl P., Chantre G.R., González-Andujar J.L. (2021). IPMWise, a Decision Support system for Multispecies Weed Control in Cereal Crops. Decision Support Systems for Weed Management.

[45] Milani A., Panozzo S., Farinati S., Iamonico D., Sattin M., Loddo D., Scarabel L. (2021). Recent Discovery of *Amaranthus palmeri* S. Watson in Italy: Characterization of ALS-Resistant Populations and Sensitivity to Alternative Herbicides. Sustainability.

[46] Torra J., Royo-Esnal A., Romano Y., Osuna M.D., León R.G., Recasens J. (2020). *Amaranthus palmeri* a New Invasive Weed in Spain with Herbicide Resistant Biotypes. Agronomy.

[47] Chaudhari S., Jordan D., York A., Jennings K.M., Cahoon C.W., Chandi A., Inman M.D. (2017). Biology and management of glyphosate-resistant and glyphosate-susceptible Palmer amaranth (*Amaranthus palmeri*) phenotypes from a segregating population. Weed Sci..

[48] Manicardi A., Milani A., Scarabel L., Mora G., Recasens J., Llenes J.M., Montull J.M., Torra J. (2023). First report of glyphosate resistance in an *Amaranthus palmeri* population from Europe. Weed Res..

[49] Tehranchian P., Norsworthy J., Powles S., Bararpour M., Bagavathiannan M., Barber T., Scott R. (2017). Recurrent Sublethal-Dose Selection for Reduced Susceptibility of Palmer Amaranth (*Amaranthus palmeri*) to Dicamba. Weed Sci..

[50] Lindsay K., Popp M., Norsworthy J., Bagavathiannan M., Powles S.B., Lacoste M. (2017). PAM: Decision Support for Long-Term Palmer Amaranth (*Amaranthus palmeri*) Control. Weed Technol..

[51] Akhter M.J., Sønderskov M., Loddo D., Ulber L., Hull R., Kudsk P. (2023). Opportunities and challenges for harvest weed seed control in European cropping systems. Eur. J. Agron..

